# Choice of Antiepileptic Drugs in Idiopathic Generalized Epilepsy: UAE Experience

**DOI:** 10.1155/2015/184928

**Published:** 2015-05-20

**Authors:** Taoufik Alsaadi, Haytham Taha, Fatema Al Hammadi

**Affiliations:** Department of Neurology, Sheikh Khalifa Medical City, Abu Dhabi 51900, UAE

## Abstract

We retrospectively reviewed the electroencephalogram (EEG) reports of patients at our EEG lab from the years 2005–2010 to identify patients referred from the epilepsy clinic, with a confirmed diagnosis of idiopathic generalized epilepsy (IGE) by EEG criteria. We sought to report our experience in UAE of how often patients with IGE are placed on nonspecific antiepileptic drugs (AEDs) before being evaluated at an epilepsy referral clinic. 109 patients with a confirmed diagnosis of IGE based on EEG criteria were identified. When initially seen, 32.11% were taking a broad-spectrum (specific) AED only, 25.69% were taking a narrow-spectrum (nonspecific) AED, and 15.59% were placed on various combinations. Of the total patients who were receiving nonspecific AEDs, 35.71% were seizure-free and 64.28% were poorly controlled accounting for “pseudointractability status.” When converted to broad-spectrum (specific) AEDs, 50% became well controlled. Furthermore, 26.6% of patients, who were previously on no AED prior to the clinic visit, became well controlled once placed on specific AED.

## 1. Introduction

Idiopathic generalized epilepsy (IGE) comprises a wide variety of epileptic syndromes that are believed to have a strong genetic basis [1] and, as a group, have the highest rate of complete seizure control with the use of broad-spectrum (specific) antiepileptic drugs (AEDs) [2]. Patients with IGE often have a family history of epilepsy that tend to present during childhood or adolescence, although they may not be diagnosed or begin until adulthood (adult onset IGE) [1, 3–5]. They often have normal intelligence, normal neurological examination, and normal magnetic resonance imaging (MRI) scan. The electroencephalogram (EEG) is the only definitive test to confirm the diagnosis of IGE and, when abnormal, it can be very characteristic of the syndrome, showing generalized spikes and polyspike complexes of 3-4 Hz, or faster frequency, superimposed on a normal EEG background [6–8]. In general, IGEs respond well to treatment, with 70–80% being fully controlled. However, not all AEDs are equally effective in treating IGE. The use of narrow-spectrum (nonspecific) AEDs, such as carbamazepine (CBZ) and phenytoin (PHT), either in monotherapy or in combination, is a common wrong practice, which could account for the seemingly difficult to control seizures “pseudointractability” in some reported series [1, 9–11].

## 2. Methods

We retrospectively reviewed the EEG reports of all patients seen at our EEG lab in the period from the years 2005–2010. Patients with EEG criteria consistent with a diagnosis of IGE and referred from the epilepsy clinic at SKMC were identified. For those identified patients, we reviewed their charts, demographic data, workup for epilepsy, age of onset, seizure types, seizure frequency, and their history of AED use, prior to their evaluation at a specialized epilepsy clinic. This clinic was established in mid June 2006, with the objective of providing a comprehensive evaluation for patients with refractory epilepsy. The clinic is managed by an epileptologist along with other neurologists and supportive staff. We recorded the seizure response rate based on the patients' last 6 months clinic visits and compared it to a 6-month period following their evaluation at the epilepsy clinic and initiation of the “broad-spectrum” AED, if indicated. We have divided the types of AED use into broad-spectrum (specific) and narrow-spectrum (nonspecific). It is well established that certain AEDs are more specific than the others for the treatment of IGE, namely, valproate (VPA), lamotrigine (LTG), topiramate (TPM), and levetiracetam (LEV) [10–16]. On the other hand, the group of “nonspecific” AEDs include phenytoin (PHT), carbamazepine (CBZ), oxcarbazepine (OXC), and gabapentin (GBP). We have used the International League Against Epilepsy 1989 classification to classify the different epilepsy types [17, 18].

The primary objective of our study was to report our experience in UAE of how often patients with IGE are misdiagnosed and/or mistreated with nonspecific AEDs prior to being evaluated by the epilepsy clinic. The secondary objective was to determine the percentage of patients who become adequately controlled after evaluation at the epilepsy clinic and switched to the “right” choice of AEDs.

## 3. Results

109 patients were identified, 50 males and 59 females, aged 12–56 with mean age of 26 and mean seizure duration of 10 years (Table 1). According to the International League Against Epilepsy classification, 89 patients (81.65%) had idiopathic generalized epilepsy, 17 patients (15.59%) had juvenile myoclonic epilepsy (JME), and 3 patients (2.75%) had juvenile absence epilepsy (JAE) (Table 2).

When initially seen, 29 patients (26.6%) were not on any AED, and 35 patients (32.11%) were using specific AED (Table 3); of those, 62.85% were on VPA, 8.57% were on TPM, 8.57% were on LTG, 8.57% were on LEV, and 11.43% were on various combinations of specific AEDs (Table 4). On the other hand, 28 patients (25.69%) were taking narrow-spectrum (nonspecific) AEDs (Table 3); of those, 53.57% were on CBZ, 10.71% were on PHT, 3.57% were on GBP, 3.57% were on PB, 3.57% were on OXZ, and 25% were on various combinations of these nonspecific AEDs (Table 5). The remaining 17 patients (15.59%) were on a combination of both specific and nonspecific AEDs (Table 3).

Of the total 28 patients who were receiving nonspecific AEDs, seizures were adequately controlled in 10 patients (35.71%), while 18 patients (64.28%) had poorly controlled seizures (Table 6). When these patients' AED regimens were changed from nonspecific to a specific AED, 14 patients (50.0%) became fully controlled, 8 patients (28.57%) appeared to be truly intractable to all medication regimens, and 6 patients (21.42%) have missed followup (Table 6).

## 4. Discussion

To our knowledge, this is the first study in the Middle-East region that demonstrates the percentage of IGE patients who seemingly have difficult to control seizures (pseudointractable), but, in reality, they were using “nonspecific” AEDs. Our findings underscore the importance of establishing accurate diagnosis based on syndromic classification. As a matter of fact, the International League Against Epilepsy explicitly recommends that the classification of syndromes be “used daily in communication between colleagues” and be the “subject of clinical trials and other investigations.”

Our findings in our region are similar to other series, where 30% of patients with IGE were on nonspecific medications and 65% of them had poorly controlled seizures. When switched to more specific AEDs, 50% became seizure-free. This shows the importance of thorough and comprehensive evaluation of patients with difficult to control seizures before they are deemed refractory to AEDs. Interestingly, however, 34% of IGE patients treated with “nonspecific” drugs, such as CBZ or PHT, were seizure-free. Of note, all these patients had GTCs as the predominant seizure type, and none of them had associated absence or myoclonic seizures. It is well established that these latter seizure types may worsen with the use of certain AEDs, whereas GTCs may respond well to a narrow-spectrum (nonspecific) AEDs [2, 11, 16].

Our study has clinical implications as most patients with generalized tonic clonic (GTC) seizures are assumed to have focal seizures with secondary generalization, especially if their seizures start in adult life [19]. Indeed, 22% of our IGE patients had their seizures beginning after the age of 20. This emphasizes the need to keep an open mind approach, when evaluating these patients, and to consider using broad-spectrum AEDs if in doubt about the underlying syndromic diagnosis.

We realize that our study has its limitations. It is relatively small, single center, and retrospective. Some patients were missed to follow-up. Moreover, it has a selection bias, as specialty epilepsy clinics tend to evaluate patients that are doing poorly. Indeed, and as illustrated in our cohorts, a significant proportion of our patients were doing poorly at the time of referral, and 28.57% of them have remained intractable despite a trial of several AEDs.

## 5. Conclusion

Our findings confirm the previous views that a poor choice of AED is still the main cause of IGEs that are seemingly difficult to control and show the importance of establishing specialized epilepsy clinics to evaluate these patients and make the appropriate changes. In our region, the inappropriateness of some AEDs for IGE is still not well recognized in a significant proportion of our patients.

## Figures and Tables

**Table 1:** Patient demographics.

Total number	109
Mean age	26
Male	50
Female	59
Duration of seizures (mean)	10 years
Age of onset (mean)	16
Age >20	24 (22.01%)
EEG (IGE alone)	96 (88.07%)
EEG (IGE + Focality)	13 (11.92%)
Family History of seizures, excluding febrile Sz	17 (15.59%) 1st degree relatives, 5 (4.58%) 2nd degree relatives

**Table 2:** Epilepsy/seizure types.

Epilepsy type	Seizure types	Total patients
Idiopathic generalized epilepsy with generalized tonic clonic seizures	(89) 100% GTCs	89 (81.65%)

Juvenile myoclonic epilepsy	15 (88%) GTCs 17 (100%) myoclonic	17 (15.59%)

Juvenile absence epilepsy	3 (100%) GTCS 3 (100%) absences	3 (2.75%)

**Table 3:** Prior AED use.

Patients on no prior AED	29 (26.60%)
Patients on specific AED	35 (32.11%)
Patients on nonspecific AED	28 (25.69%)
Patients on combination of specific and nonspecific AED	17 (15.59%)

**Table 4:** Prior adequate AED use.

Patients on specific AED	35 patients
(1) Valproate	22 (62.85%)
(2) Topiramate	3 (8.57%)
(3) Lamotrigine	3 (8.57%)
(4) Levetiracetam	3 (8.57%)
(5) Combination	4 (11.43%)

**Table 5:** Prior nonspecific AED use.

Patients on nonspecific AED	28 patients
(1) Carbamazepine	15 (53.57%)
(2) Phenytoin	3 (10.71%)
(3) Gabapentin	1 (3.57%)
(4) Phenobarbital	1 (3.57%)
(5) Oxcarbazepine	1 (3.57%)
(6) Combination	7 (25.0%)

**Table 6:** Treatment response in nonspecific AED group.

28 patients	Prior nonspecific AED	Change adequate AED
Adequately controlled seizures	10 (35.71%)	14 (50.0%)

Poorly controlled seizures	18 (64.28%)	8 (28.57%)

Missed to follow up		6 (21.42%)
